# The Influence of Preparation Design in Biologically Oriented Preparation and Horizontal Preparation on Tooth Structure Removal: An In Vitro Study

**DOI:** 10.1002/cre2.70487

**Published:** 2026-10-25

**Authors:** Abeer Aljareh, Nabil Alhouri, Mohanad Kadhim

**Affiliations:** ^1^ Department of Fixed Prosthodontics Faculty of Dentistry, Damascus University Damascus Syria; ^2^ Department of Periodontology Faculty of Dentistry, Warka University College Basra Iraq

**Keywords:** biologically oriented preparation, structure removal, tooth preparation, zirconia crowns

## Abstract

**Objectives:**

Several clinical scenarios indicate the need for full‐coverage crowns. However, these restorations require substantial tooth reduction, which may compromise pulpal vitality and biological principles. This study aimed to evaluate the effect of the biologically oriented preparation technique on the volume and weight of tooth structure removal compared with horizontal preparation when zirconia crowns are used.

**Material and Methods:**

A total of 160 acrylic teeth representing the maxillary right central incisor, canine, premolar, and molar, were divided into two main groups according to preparation type: biologically oriented preparation with no finish line (*n* = 80), and horizontal preparation with a 1‐mm rounded shoulder (*n* = 80). Each group was further subdivided into a monolithic zirconia crown subgroup (*n* = 40) and a bilayered zirconia crown subgroup (*n* = 40). Teeth were weighed and scanned before preparation. All preparations were performed by a single operator following standardized guidelines. After preparation, the teeth were reweighed and rescanned. The percentage of tooth structure removed was calculated by weight, and the scanned files were analyzed to determine the volume of removed tooth structure.

**Results:**

A significant difference was observed in the amount of tooth structure removal between the biologically oriented preparation (32.33%) and horizontal preparation groups (41.48%). Bilayered zirconia crowns required significantly more reduction than monolithic crowns (42.55% and 31.26%, respectively). The tooth structure removal also differed significantly according to tooth type. Furthermore, a high degree of interchangeability was found between the two assessment methods, demonstrated by a strong correlation (r = 0.99) and a mean difference of 2.8%.

**Conclusions:**

Within the limitations of this study, the biologically oriented preparation technique may represent a more conservative alternative to the horizontal shoulder preparation, particularly when paired with monolithic zirconia crowns rather than bilayered zirconia restorations.

## 1 Introduction

During function, natural teeth are exposed to various factors that may lead to the loss of dental tissues, including caries, trauma, and tooth wear. When such conditions occur, restorative interventions, including the placement of full coverage crowns, become necessary (Weber et al. 2022). Although the preservation of tooth structure is a fundamental principle of tooth preparation (Shillingburg et al. 2012), a sufficient amount of reduction is still required to meet esthetic demands and to ensure the long‐term durability of the restoration (Shillingburg et al. 2012; Goodacre et al. 2001; Monaco et al. 2019).

Horizontal preparation, particularly the rounded shoulder design, is widely regarded as a reliable approach for ceramic restorations, as it provides sufficient support, adequate fit, and stability for ceramic crowns (Shillingburg et al. 2012; Alqahtani et al. 2025). However, vertical preparation has emerged as a more conservative alternative (Kasem et al. 2023) while still demonstrating comparable fracture resistance in all‐ceramic restorations (Cortellini et al. 2015). The biologically oriented preparation technique (BOPT) has been introduced as a type of vertical preparation that may offer several biological and clinical advantages (Łabno and Drobnik 2020), including the preservation of tooth structure (Loi and Di Felice 2013). As such, it might align with the main objective of dentistry, which is to conserve as much healthy tissue as possible (Loi and Di Felice 2013). BOPT differs from conventional preparation in multiple respects. Most notably, BOPT eliminates the emergence profile of the anatomical crown by creating an axial vertical plane between the tooth root and crown (Agustín‐Panadero et al. 2021), thereby enabling the prosthodontic crown to replicate the natural tooth emergence profile (Agustín‐Panadero et al. 2021). In addition, BOPT involves a finish area rather than a defined finish line (Loi and Di Felice 2013), permitting crown margins to be positioned 0.5–1 mm within the gingival sulcus without invading the biological width (Loi and Di Felice 2013; Agustín‐Panadero et al. 2021; Serra‐Pastor et al. 2019).

Metal‐ceramic crowns have traditionally been considered a reliable restorative option (Edelhoff and Sorensen 2002a). Nevertheless, they require substantial tooth reduction; up to 71.9% of the coronal structure in anterior teeth and as much as 75.6% in posterior teeth (Edelhoff and Sorensen 2002a; Edelhoff and Sorensen 2002b). All‐ceramic crowns are regarded as highly esthetic restorative options due to their translucency (Monaco et al. 2019). The earliest application of zirconia in fixed prosthodontics involved the fabrication of high‐strength zirconia cores veneered with feldspathic porcelain (Abduo et al. 2023). However, these bi‐layered restorations were prone to frequent chipping of the veneering ceramic in spite of their high esthetic properties (Abduo et al. 2023; Pontevedra et al. 2022). The introduction of monolithic all‐ceramic crowns has helped reduce such complications by eliminating the weak veneer layer (Monaco et al. 2019; Fasbinder et al. 2010). In addition to improved mechanical reliability, monolithic restorations may allow for more conservative tooth preparation compared with bilayered all‐ceramic crowns (Monaco et al. 2019; Schwindling et al. 2019). Yet, the limited ability to individualize their shape and shade remains a key drawback of monolithic zirconia (Abduo et al. 2023).

Several methods have been utilized to appraise dental structure removal during different tooth preparation designs. Reported values vary depending on the assessment method employed (Weber et al. 2022). For instance, gravimetric analysis has shown that full‐crown preparations require tooth structure removal of approximately 40.00%–45.01% (Monaco et al. 2019), whereas volumetric analysis using micro‐computed tomographic imaging has yielded higher values, ranging from 49.84% to 65.26% (Al‐Fouzan and Tashkandi 2013). Moreover, some studies have assessed the impact of tooth preparation by measuring residual dentine thickness as an indicator of structural preservation (Adu‐Ampomah et al. 2023; Borelli et al. 2013).

Numerous studies have examined the amount of tooth structure removal due to different preparation designs for full crowns made of diverse materials. Weber et al. (2022) reported that shoulder preparation required a structure removal of 28.73%–33.74% by volume and 29.48%–34.40% by weight in different teeth (Weber et al. 2022). Comparable percentages were observed during posterior teeth preparations for various crowns in another investigation (Monaco et al. 2019). It has also been indicated that vertical preparation with a knife‐edge finish line, which differs from BOPT, is less invasive in terms of dental structure removal than horizontal preparation (Monaco et al. 2019). In contrast, preparation of posterior teeth for zirconia crowns was found to involve substantially less reduction, approximately 17.89%–20.65% by weight when compared to lithium disilicate and metal‐ceramic crowns (Ebrahimpour and Toronto 2017). Despite these insights and the growing clinical interest in the BOPT (Loi and Di Felice 2013; Agustín‐Panadero et al. 2021; Serra‐Pastor et al. 2019), only one *in vitro* study has investigated the effect of BOPT on the volume of tooth structure removal when compared to chamfer and modified BOPT which extends 2 mm in the gingival sulcus until the bone (Real‐Voltas et al. 2025). However, there is a lack of specific evidence on the influence of preparation design included in the BOPT on the weight and volume of structure removal for all‐ceramic crowns. Therefore, this study aimed to evaluate the amount of tooth structure removal in BOPT and horizontal shoulder preparation for monolithic and bilayered zirconia crowns using weight and volume analyses. The primary null hypothesis is that there was no difference in the amount of tooth structure removal between BOPT and horizontal preparation for monolithic and bilayered zirconia crowns, both volumetrically and gravimetrically. The secondary null hypothesis is that neither crown type nor tooth type had an effect on the amount of structure removal due to preparation.

## 2 Materials and Methods

This study was conducted entirely at the Faculty of Dentistry at Damascus University and was formally registered as part of a larger research project. The study was approved by Damascus University's Ethics Committee under record number (DN‐261124‐353).

A total of 160 maxillary right acrylic teeth of different morphologies (central incisor, canine, second premolar, and first molar; Kilgore, USA) were utilized in this study. The required sample size was calculated using G*Power software (V 3.1.9.4; Heinrich Heine University Düsseldorf, Germany) (Faul et al. 2007) using *t*‐test for the difference between two independent means. The effect size was calculated from the mean and standard deviation of two groups in a previous study (Monaco et al. 2019). Power analysis indicated 10 specimens per group by applying 95% statistical power at a 5% significance level and the calculated effect size was 1.6. The teeth were randomly allocated into two preparation groups: BOPT and conventional horizontal preparation (*n* = 20 per tooth morphology) (Figure 1). Each preparation group was further subdivided according to the type of all‐ceramic crown—monolithic zirconia or fully veneered zirconia (bilayered zirconia)—with 10 specimens assigned to each subgroup. All samples were stored in numbered boxes throughout the experimental procedures.

**Figure 1:**
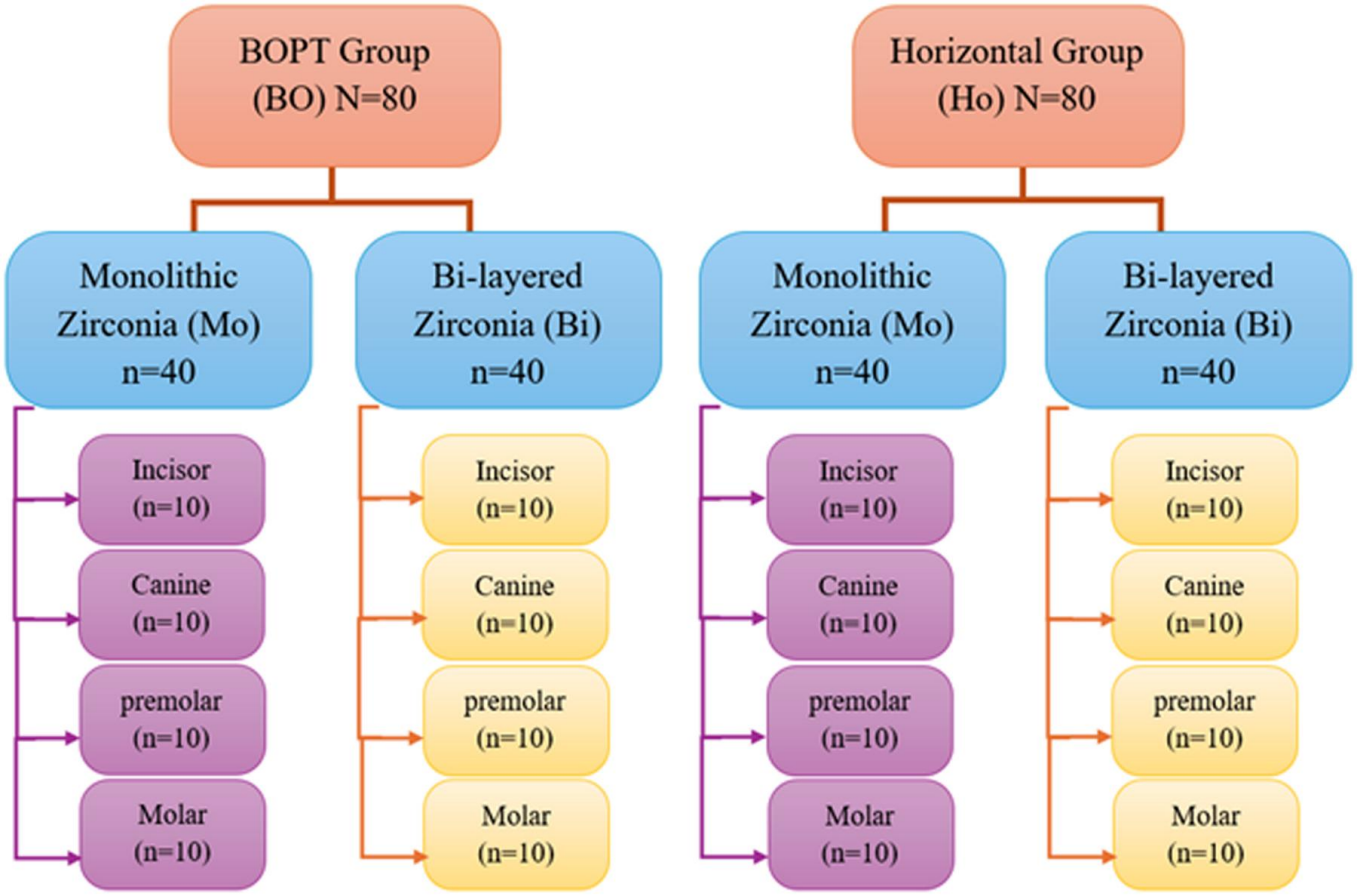
Study groups and subgroups.

### 2.1 Calibration and Intra‐Operator Reliability

Prior to the definitive study, the operator, who is an expert in fixed prosthodontics, prepared numerous teeth according to the studied variables. Then, they underwent a calibration phase consisting of preparing 32 pilot teeth to evaluate intra‐operator reliability, divided into 16 teeth per week. A 1‐week wash‐out period was implemented between the preparation sessions. This time interval was selected to eliminate any recall effect regarding the baseline tooth anatomy from the first session, while ensuring that the environmental conditions and equipment performance remained constant. These teeth were weighed using a high‐precision balance (Sartorius BP 110 S, Max 110 g, Germany) with an accuracy of 0.0001 g, before and after preparation to verify the consistency of tooth structure removal. Data normality was verified using the Shapiro–Wilk test, which confirmed a normal distribution (*p* > 0.05). To assess intra‐operator reliability and check for potential systematic bias between the two preparation sessions, a paired‐samples Student's *t*‐test was conducted. The analysis revealed no statistically significant difference in the percentage of tooth structure removal between Week 1 and Week 2 (*p* = 0.414). This statistical evidence demonstrates high reproducibility and good intra‐operator calibration. Four teeth from the pilot study were scanned after preparation to verify the stability of the convergence degree using Geomagic Control X software (V 2022 0.1, Hexagon AB, Stockholm, Sweden), using properties of the cone tool as described subsequently. The convergence degrees data exhibited a normal distribution according to the Shapiro–Wilk test (*p* > 0.05), and the paired‐samples Student's *t*‐test showed no difference in the taper degree (*p* = 0.31), indicating good intra‐operator reliability regarding both preparation consistency and taper stability.

#### 2.1.1 Initial Weighing and Scanning

In order to determine the weight of the coronal portion, an additional unprepared tooth of each morphology was employed. Because the mean weights of the additional teeth and the experimental teeth fell within the same range (±0.0001 g), it was possible to use these additional teeth rather than the studied ones for this procedure. The cementoenamel junction (CEJ) was identified, and a reference line was marked 2 mm apical to the CEJ. The root was sectioned at this reference line using a 0.3‐mm‐thick disc mounted on a low‐speed handpiece, after which the separated root was weighed three times using the high‐precision balance, and the mean value was calculated. This value was then subtracted from the weight of the experimental teeth (both before and after preparation) to obtain the weight of the coronal portion and the 2 mm radicular segment for the gravimetric analysis.

All teeth were cleaned with 95% ethyl alcohol and subsequently dried using compressed air (Edelhoff and Sorensen 2002a; Sadid‐Zadeh et al. 2018). The initial weight of each specimen was measured three times using the high‐precision balance, and the corresponding mean value—after subtracting the weight of the radicular part—was recorded as the pre‐preparation tooth weight. To ensure experimental accuracy, the balance was recalibrated after every five measurements.

Prior to preparation, the CEJ was marked with a pencil. Then, resin composite buttons were placed on the mesial and distal root surfaces, positioned 3 mm apical to the CEJ. These buttons served as reference markers during the alignment process in the volumetric study. The teeth were embedded by their roots into the paste of the scanner base. Then, they were scanned prior to preparation using a laboratory scanner (BlZ LS200, Wuhan, China), with an accuracy of ≤ 8 µm, and the resulting digital files were imported into an STL (Standard Tessellation Language) format to serve as baseline references for the volumetric analysis.

### 2.2 Tooth Preparation

All teeth were prepared by a single, expert, calibrated operator, while a second expert operator verified the preparations. Preparations were carried out under dry conditions using a high‐speed handpiece (Apple Dental A2‐TUP, Jinme Medical Technology Co. Inc., China). To avoid the effect of heat and to clean the tooth during preparation, 95% ethyl alcohol was sprayed repeatedly. Silicone jigs (Zetaplus, Zhermack, Italy) were applied as supportive bases to stabilize the teeth during preparation. Additionally, silicone indices in combination with a Williams periodontal probe (Derby, Massarosa, Italy) were employed to assess and verify the amount of tooth reduction all through the preparation procedures (Hussain et al. 2007) (Figure 2a). A millimetric ruler was used to verify the degree of convergence, too (Figure 2b).

**Figure 2:**
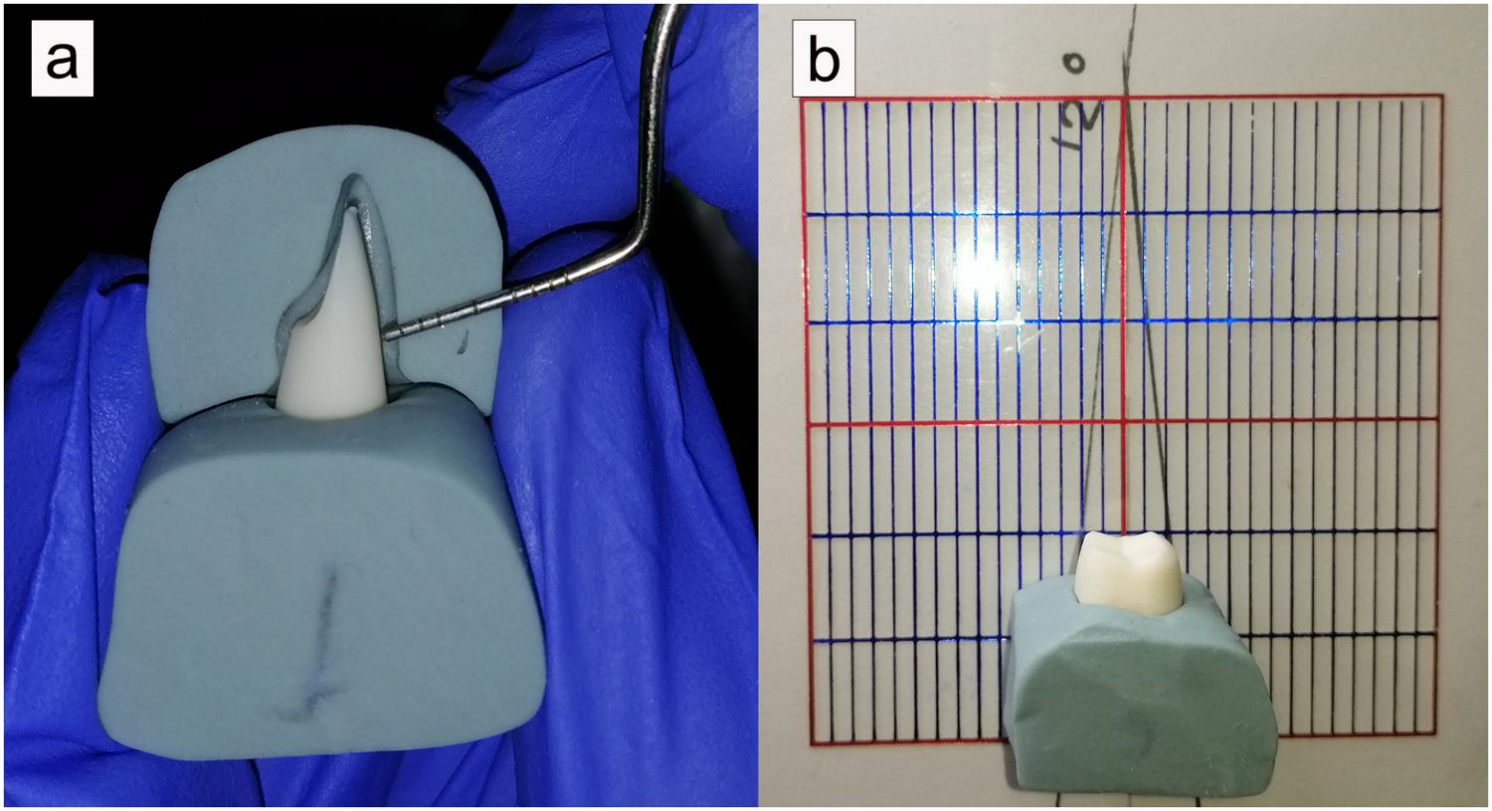
Silicone index used to assess the amount of tooth reduction (a). Millimetric ruler used to check the taper (b).

The occlusal surfaces of all teeth were prepared using guide grooves with a depth equivalent to half the diameter of the straight fissure bur. The occlusal and incisal surfaces of teeth in the bilayered crown groups required an additional 0.5 mm of tooth reduction compared with the monolithic crown groups. The occlusal reduction was verified during the preparation by the silicone index and the periodontal probe.

In the horizontal preparation group, a rounded shoulder with a depth of 1 mm was prepared 0.5 mm coronal to the CEJ. Guide grooves, prepared at half of the tapered diamond bur's diameter, guided the axial reduction in this group. The silicone index and the periodontal probe were used to verify the reductions, while the probe was also utilized to assess the width of the shoulder finish line. In the BOPT group, teeth were prepared in three steps, guided by the angulation of the bur relative to the long axis of the tooth, adhering to the protocol described in previous studies (Loi and Di Felice 2013; Serra‐Pastor et al. 2019). The long flame‐shaped diamond bur was initially positioned at a 10° angle against the tooth long axis, using its tip to eliminate the marked CEJ. It was then aligned parallel to the tooth's long axis for axial reduction extending 0.5 mm apical to the CEJ, and finally adjusted to approximate 5° angle to achieve the appropriate taper. Table 1 shows the reduction values for each group alongside the corresponding burs utilized. During and after each step, the reduction was checked by the periodontal probe and the silicone index. The finish area consistency was assessed by a sharp dental explorer moved vertically throughout the axial surfaces from the coronal part to the root surface. The CEJ elimination was evaluated by erasing the mark line. The prepared surfaces were refined and line angles were rounded using fine diamond burs (Ebrahimpour and Toronto 2017). The convergence degree and reduction were finally rechecked (Figure 2). During and after the preparation procedures, teeth were cleaned with 95% ethanol and subsequently stored in their numbered boxes (Figure 3).

**Table 1:** Summary of the tooth reduction amount and the burs employed for preparation.

Preparation type	Crown type	Reduction	Burs
BOPT	Monolithic	Axial	Long flame‐shaped diamond bur (ISO 229/0.13, FO‐11, LusterDent, France) Flame bur (ISO 298/106, FO‐22, Mani, Japan) for palatal surface in anterior teeth
		1 mm with no finish line (Stevens et al. 2024; Omnia et al. 2022). (CEJ was eliminated)	
	Bilayered	1.5 mm with no finish line (Monaco et al. 2017). (CEJ was eliminated)	
Horizontal	Monolithic	1 mm with 1 mm‐rounded shoulder finish line (Stevens et al. 2024; Omnia et al. 2022).	Tapered diamond bur (ISO 173/0.18, TF‐12, Mani, Japan) Flat‐end slightly rounded tapered fine diamond bur (ISO 845/012, 845F‐012, Mani, Japan) Flame bur (ISO 298/106, FO‐22, Mani, Japan) for palatal surface in anterior teeth
	Bilayered	1.5 mm with 1 mm‐rounded shoulder finish line (Monaco et al. 2017).	
Both groups	Monolithic	Occlusal	Straight fissure diamond bur (ISO 109/013, SF‐HISC, Mani, Japan)
		1 mm (nonfunctional cusps)‐ 1.5 mm (functional cusps and incisal edge in interior teeth) (Stevens et al. 2024; Omnia et al. 2022).	
	Bilayered	1.5 mm (nonfunctional cusps)‐ 2 mm (functional cusps and incisal edge in interior teeth) (Monaco et al. 2017).	

**Figure 3:**
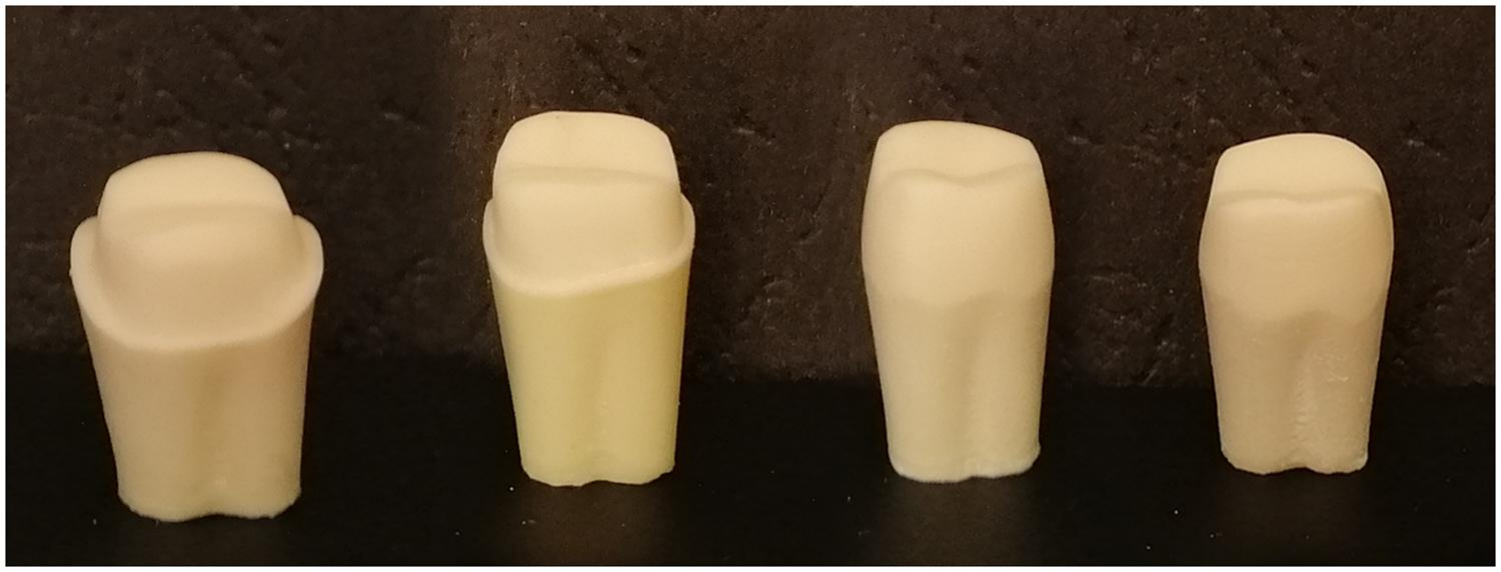
Prepared maxillary molars (preparation type–crown type) from left to right; horizontal‐bilayered, horizontal‐monolithic, BOPT‐bilayered, and BOPT‐monolithic.

After preparation, all teeth were checked by the external operator. Moreover, all teeth were rescanned and the taper was re‐verified using the digital approach described above to assess intra‐operator stability. In this approach, prepared teeth were scanned and the STL files were imported into Geomagic Control X software. The convergence degree was assessed by adding a cone guided by the axial walls of the studied teeth. Subsequently, the taper was calculated using the half‐angle of the cone. The acceptable convergence degree was 6° ± 0.85° (Figure 4a,b). Moreover, each studied prepared tooth was aligned to a standard prepared tooth using the best‐fit alignment tool, with the composite buttons serving as reference markers for the alignment. The 3D Compare tool was then utilized to verify the overall preparation, Figure 4c.

**Figure 4:**
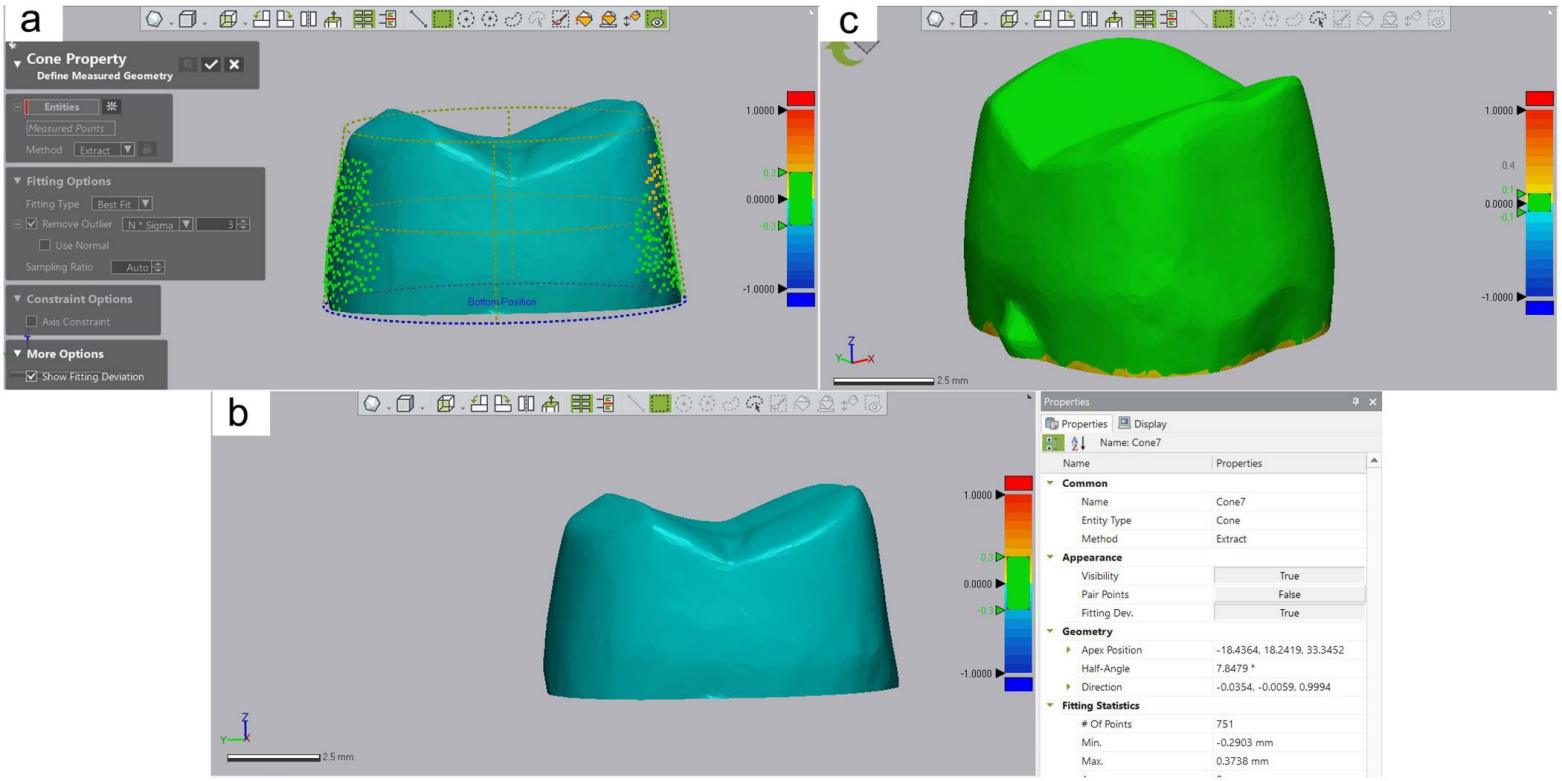
Verification process: cone tool insertion (a), taper calculation from cone properties (b), and alignment of a prepared tooth with a reference prepared tooth to check the overall preparation (c).

### 2.3 Volumetric Analysis

Following preparation, the corresponding STL files of the scanned specimens were generated and imported into the software for comparative analysis. Reverse engineering software Geomagic Design X (V 2022 0.0, Hexagon AB, Stockholm, Sweden) was employed to process the digital scan files of each tooth before and after preparation. The prepared tooth was aligned with its corresponding reference (unprepared tooth) using the interactive alignment tool, with resin composite buttons serving as reference markers during the alignment process, Figure 5. A plane was established 2 mm apical to the CEJ, and the radicular portion was sectioned using the split‐to‐plane tool. The volume of the coronal portion of each tooth, both before and after preparation, was calculated using the volume function of the software. The percentage of tooth structure removal was determined according to the following formula:

$$\mathrm{RSv}=\frac{V_{0}-V}{V_{0}}*100,$$

where *V*_0_ represents the volume of the coronal portion of the tooth prior to preparation, *V* is the volume of the coronal portion after preparation, and *RSυ* indicates the percentage of tooth structure removal.

**Figure 5:**
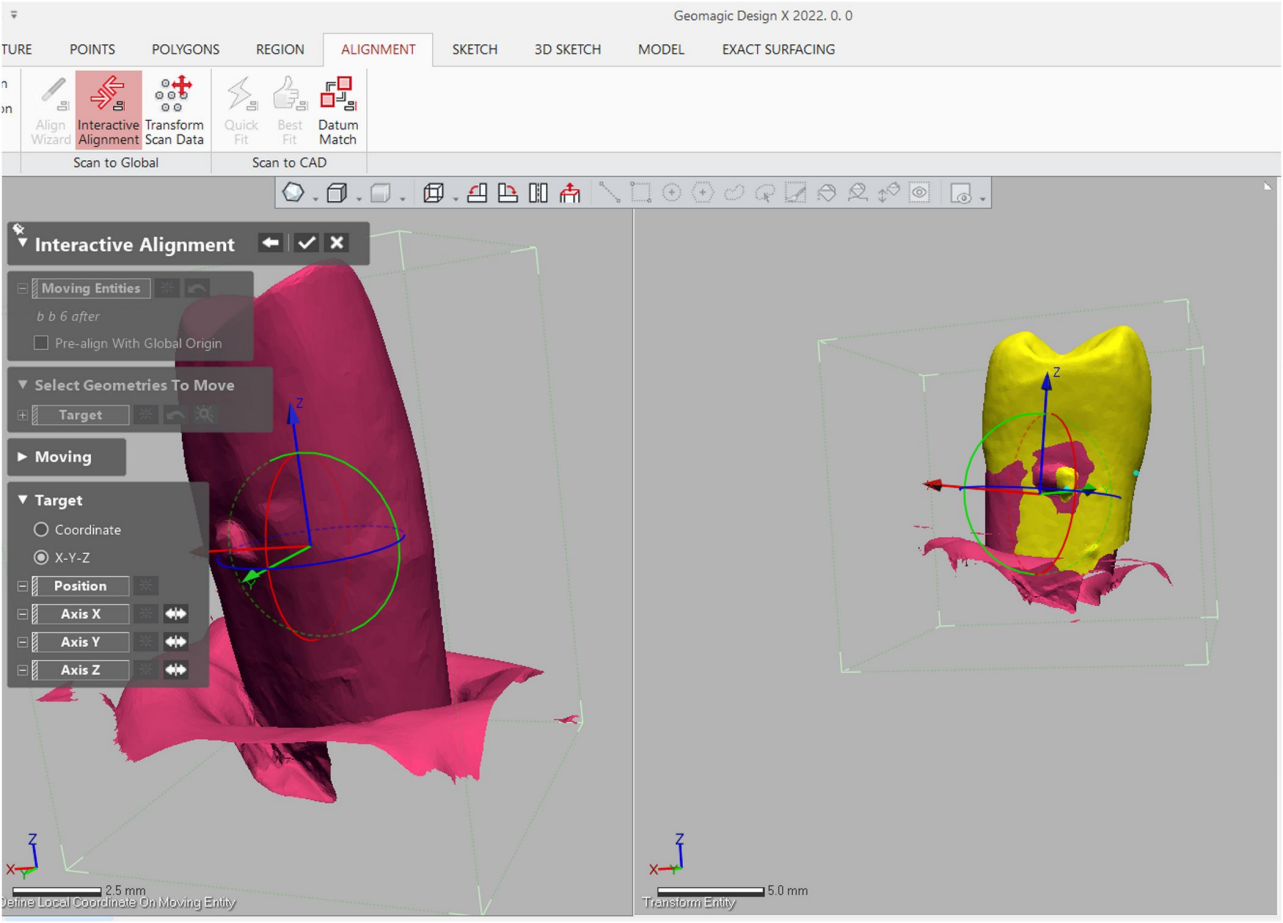
Alignment of the tooth before and after preparation using resin composite buttons as reference markers.

### 2.4 Gravimetric Analysis

After scanning, the composite buttons were removed and the areas of the buttons were checked by a sharp dental explorer. The teeth were cleansed with ethyl alcohol and gently air‐dried. Subsequently, each tooth was weighed three times using the high‐precision balance. The mean of these measurements was recorded, and the root weight was subtracted accordingly. The percentage of tooth structure removal was then calculated using the formula:

$$\mathrm{RS}=\frac{W_{0}-W}{W_{0}}*100,$$

where W_0_ represents the weight of the unprepared coronal portion of the tooth, *W* is the weight of the prepared coronal portion, and RS indicates the percentage of tooth structure removal (Monaco et al. 2019; Edelhoff and Sorensen 2002a; Edelhoff and Sorensen 2002b).

### 2.5 Statistical Analysis

Statistical analysis was performed using IBM SPSS Statistics 27 (SPSS, Chicago, IL, USA) and JASP software (version 0.96, Netherlands). The Shapiro–Wilk test indicated that the percentages of tooth structure removal, measured using gravimetric and volumetric methods, were not normally distributed across tooth type, preparation type, and monolithic crown groups (*p* < 0.05). Furthermore, Levene's test indicated that the homogeneity of variances could not be assumed (*p* < 0.05). Consequently, a three‐way analysis of variance (ANOVA) was performed with a Bonferroni adjustment. Estimated marginal means were calculated, and post hoc comparisons were conducted using Bonferroni correction. Spearman's rho correlation coefficient was employed to evaluate the relationship between the two measurement methods assessing structure removal, while a Bland‐Altman test was utilized to determine the interchangeability between the two methods.

## 3 Results

The horizontal preparation was associated with approximately one and one‐third times greater tooth structure removal than the BOPT group, with a significant difference between the two preparation groups in both volumetric and gravimetric measurements. The statistical test also revealed a significant difference in the amount of structure removal, by both weight and volume, between the two crown groups. The monolithic zirconia crown group was associated with significantly less tooth structure removal compared with the bilayered zirconia crown group (31.26% and 42.55% by weight, respectively) (Table 2).

**Table 2:** Three‐way ANOVA test with Bonferroni correction comparing the two preparation types, the two crown groups, and the four tooth types.

Tooth structure removal (%)	Preparation type	Marginal mean	95% CI for mean difference		SE	*t*	*df*	*p*
			Lower	Upper				
By weight	BOPT	32.33	31.47	33.18	0.378	85.58	144	0.001*
	Horizontal Prep.	41.48	40.63	42.34	0.378	109.8	144	
By volume	BOPT	28.33	27.47	29.18	0.378	74.99	144	0.001*
	Horizontal Prep.	39.86	39.00	40.72	0.378	105.5	144	
	**Crown type**							
By weight	Bilayered	42.55	41.69	43.4	0.378	112.6	144	0.001*
	Monolithic	31.26	30.41	32.12	0.378	82.76	144	
By volume	Bilayered	39.49	38.63	40.34	0.378	104.5	144	0.001*
	Monolithic	28.70	27.85	29.56	0.378	75.98	144	
	**Tooth type**							
By weight	Incisor	41.19	39.83	42.54	0.534	77.09	144	0.001*
	Canine	31.69	30.33	33.04	0.534	59.31	144	
	Premolar	41.37	40.02	42.73	0.534	77.45	144	
	Molar	33.38	32.03	34.73	0.534	62.48	144	
By volume	Incisor	38.44	37.08	39.79	0.534	71.94	144	0.001*
	Canine	29.44	28.08	30.79	0.534	55.10	144	
	Premolar	37.87	36.52	39.23	0.534	70.89	144	
	Molar	30.63	29.28	31.98	0.534	57.34	144	

Abbreviations: CI, confidence interval; df, degrees of freedom; SE, standard error; t, pooled‐error *t*‐test.

*p Value is significant. *p* values were adjusted using the Bonferroni correction.

Among tooth types, premolars and incisors exhibited the highest percentages of tooth structure removal with no significant differences between them for either assessment methods, while the lowest percentages were observed in canines and molars with no significant differences between them (Table 3).

**Table 3:** Pairwise comparisons of tooth types for the percentage of structure removal by weight and by volume.

Structures removal (%)	Tooth type		Marginal mean	SE	*t*	Cohen's *d*	*df*	*p*
By weight	Incisor	Canine	9.50	0.76	144	2.81	12.57	0.001*
		Premolar	−0.19	0.76	144	−0.06	−0.25	1.00
		Molar	7.81	0.76	144	2.31	10.33	0.001*
	Canine	Premolar	−9.69	0.76	144	−2.87	−12.82	0.001*
		Molar	−1.69	0.76	144	−0.50	−2.24	0.158
	Premolar	Molar	7.99	0.76	144	2.37	10.58	0.001*
By volume	Incisor	Canine	9.00	0.76	144	2.66	11.91	0.001*
		Premolar	0.56	0.76	144	0.17	0.74	1.00
		Molar	7.81	0.76	144	2.31	10.33	0.001*
	Canine	Premolar	−8.44	0.76	144	−2.49	−11.17	0.001*
		Molar	−1.19	0.76	144	−0.35	1.582	0.695
	Premolar	Molar	7.24	0.76	144	2.14	9.59	0.001*

Abbreviations: df, degrees of freedom; SE, standard error; t, pooled‐error *t*‐test.

*p Value is significant. *p* values were adjusted using the Bonferroni correction.

When both preparation type and crown type were considered, horizontal preparation for bilayered crowns resulted in the highest percentage of tooth structure removal, followed by BOPT for bilayered crowns, horizontal preparation for monolithic crowns, and finally BOPT for monolithic crowns (Figure 6).

**Figure 6:**
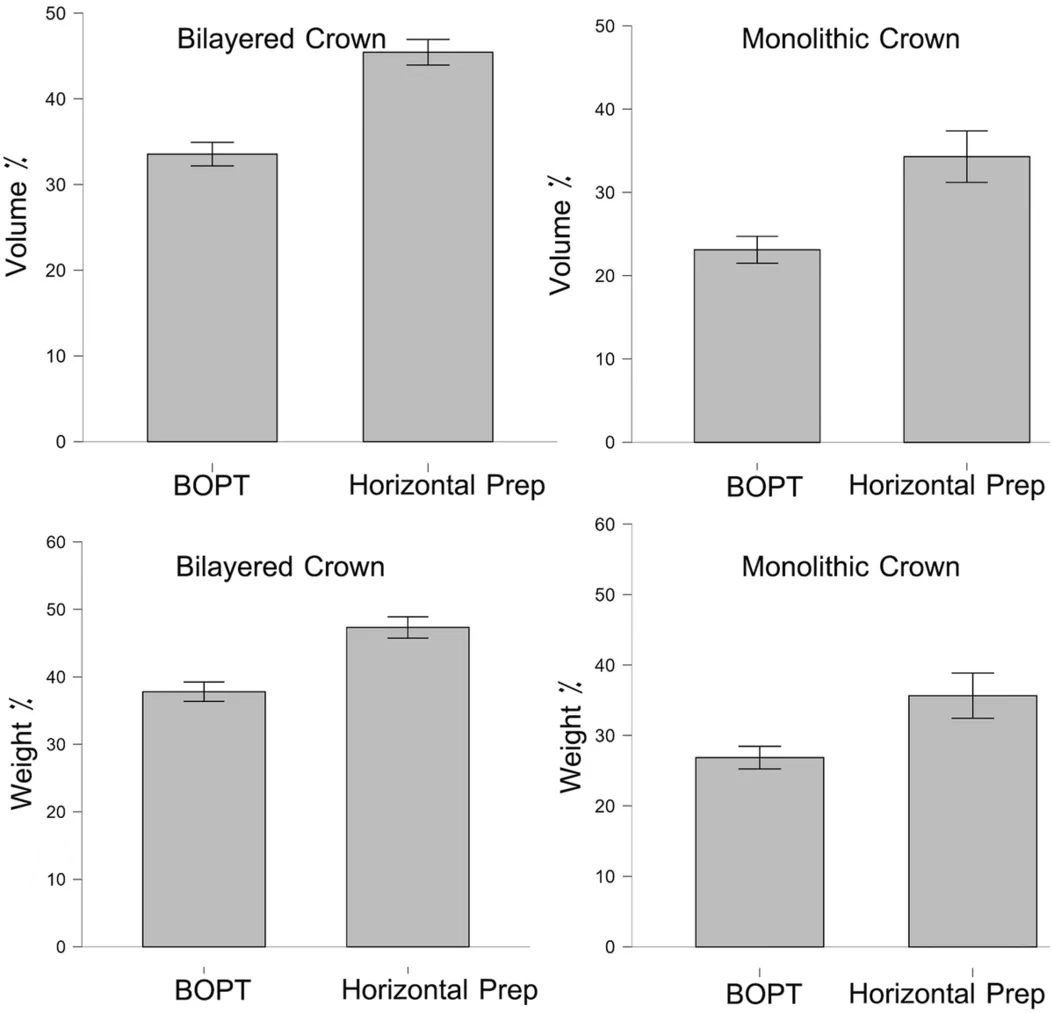
Pairwise comparison according to preparation type and crown type.

Regarding the three‐way interaction, the three‐way ANOVA with Bonferroni correction revealed a significant interaction effect (*p* < 0.001) among the three factors (preparation type, crown type, tooth type). Horizontally prepared premolars and incisors for bilayered zirconia crowns were seen to lose the highest percentage of tooth structure (49.67% and 52.46% by weight, respectively). BOPT‐prepared canines for monolithic zirconia crowns were associated with the lowest percentage of tooth structure removal (23.67% by weight, 20.67% by volume) (Figures 7 and 8).

**Figure 7:**
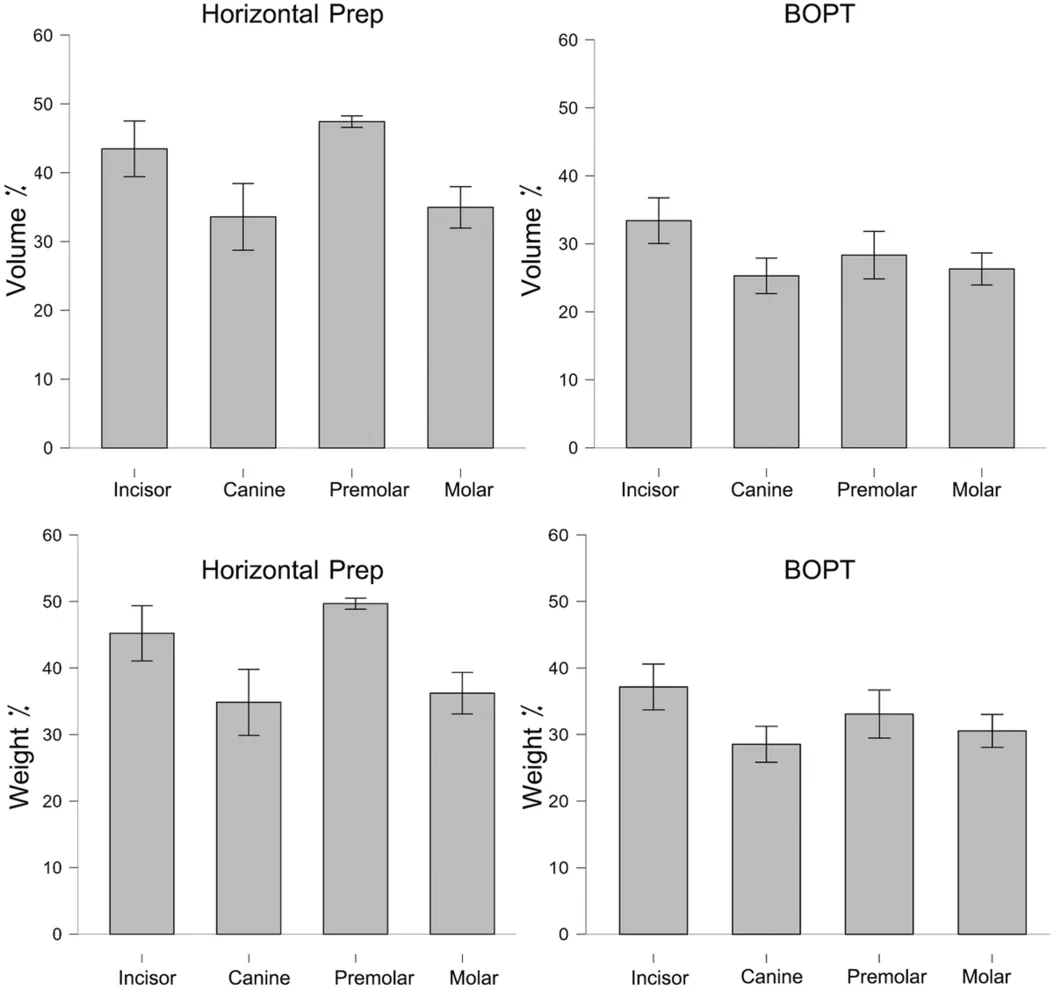
Comparison among tooth types according to preparation type.

**Figure 8:**
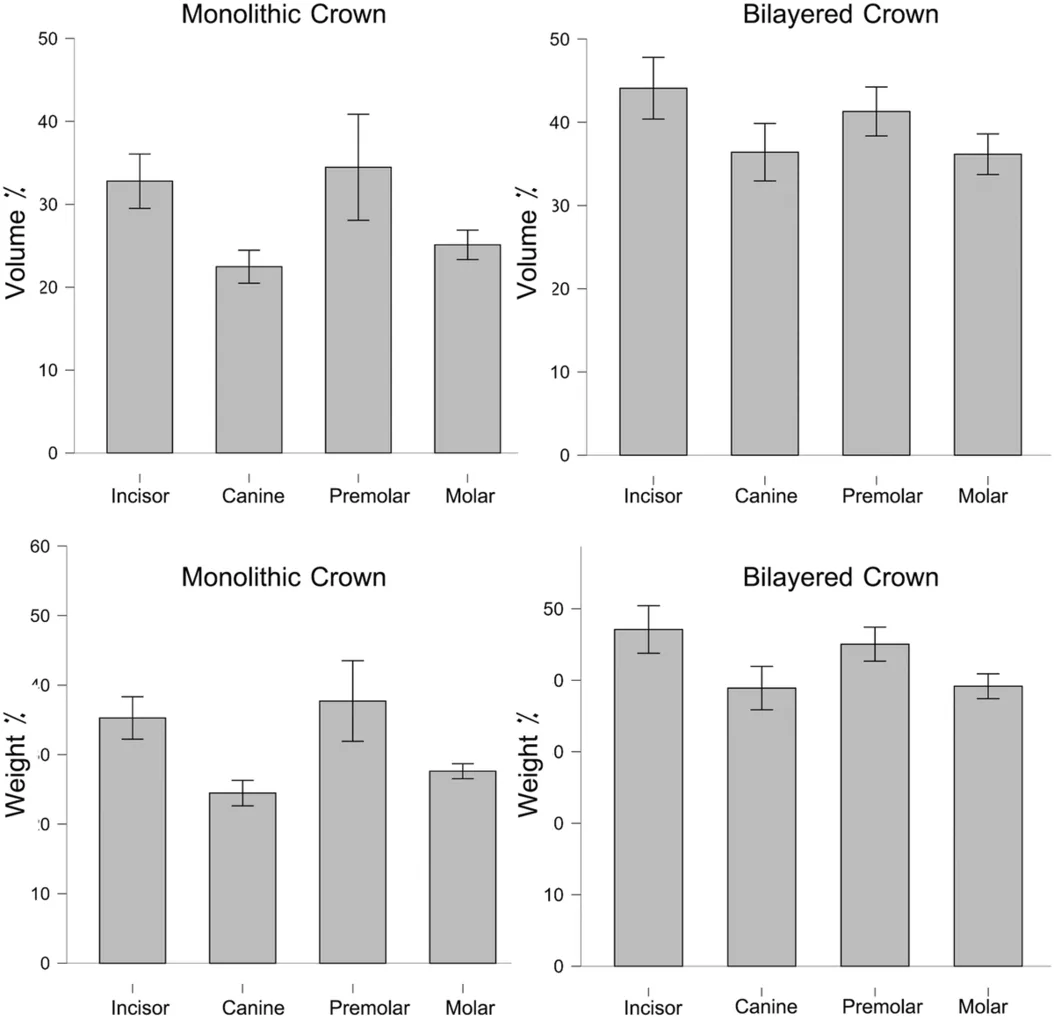
Comparison among tooth types according to crown type.

Spearman's rho demonstrated a strong positive correlation between gravimetric analysis and volumetric analysis (correlation coefficient = 0.99) (Table 4).

**Table 4:** Spearman's rho correlation evaluating the correlation between two assessment methods.

Tooth structure removal (%)		By weight	By volume
By weight	Correlation coefficient	1.00	0.99^a^
	Sig. (two‐tailed)		0.00
	*N*	160	160
By volume	Correlation coefficient	0.99^a^	1.00
	Sig. (two‐tailed)	0.00	
	*N*	160	160

a Correlation is significant at the 0.01 level (two‐tailed).

The results of the Bland–Altman analysis demonstrated a mean bias of 2.8% for the gravimetric measurements (95% CI, 2.594–3.006) with 95% limits of agreement ranging from 0.22% to 5.38% (Figure 9).

**Figure 9:**
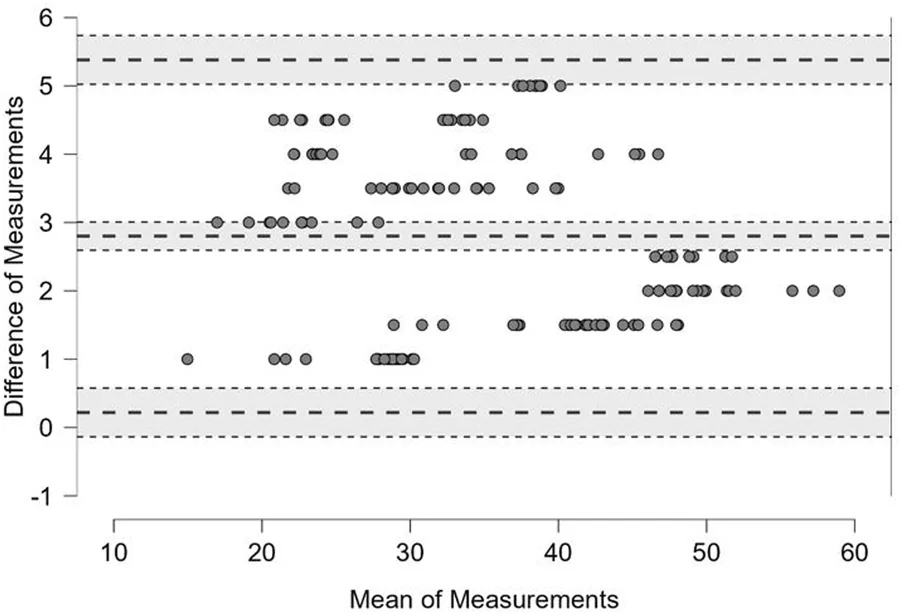
Bland–Altman test plot to assess the interchangeability of the weight and volume methods.

## 4 Discussion

The current study aimed to investigate the influence of BOPT preparation design on the amount of tooth structure removal for both monolithic and bilayered zirconia crowns in anterior and posterior teeth, compared with horizontal preparation, using two assessment methods. The results demonstrated significant differences between the two preparation designs, the two crown types, and among the various tooth groups when gravimetric and volumetric assessments were used, resulting in rejection of the null hypotheses. To strengthen the assessment, both gravimetric and volumetric measurements were used, revealing a strong correlation between the two methods. Although previous studies have examined various factors that may affect the amount of tooth structures removed due to preparation, a limited number of studies have investigated the knife‐edge vertical preparation rather than BOPT in particular (Monaco et al. 2019; Adu‐Ampomah et al. 2023; Borelli et al. 2013). Furthermore, only one study has evaluated the effect of BOPT on the volume of tooth structure removal (Real‐Voltas et al. 2025).

Acrylic teeth were used in this study to reduce the heterogeneity of natural teeth (Ebrahimpour and Toronto 2017), including differences in dentinal tubule distribution, individual morphological variation (Monaco et al. 2019), and variations in enamel and dentin density (Ebrahimpour and Toronto 2017). The homogeneity of artificial teeth also enabled the use of both volumetric and gravimetric assessment methods (Monaco et al. 2019), which were applied to comprehensively evaluate the BOPT preparation design and its effect on the amount of tooth structure removal. To replicate clinical conditions as closely as possible, the radicular portion of additional teeth was sectioned in this study as preparation is usually restricted to the coronal portion of the tooth, though it might extend 0.5 mm apical to the CEJ (Weber et al. 2022; Monaco et al. 2019). However, the roots of the experimental teeth were utilized to embed the teeth in the silicone base and scan paste, thereby stabilizing them in a manner analogous to the way the root anchors the tooth in the alveolar bone.

The results of the present study showed that horizontal preparation was associated with significantly greater tooth structure removal, approximately one and one‐third times that of BOPT, when assessed by both weight and volume. This finding can be attributed to the absence of a defined finish line in BOPT. Although BOPT preparation eliminates the CEJ during the initial phase of preparation, this step is performed using only the tip of the bur, after which the axial walls are shaped without creating a finish line. In contrast, horizontal preparation requires simultaneous axial reduction and finish line formation, thereby resulting in greater tooth structure removal. Furthermore, in this study, BOPT was compared with a 1‐mm rounded‐shoulder horizontal preparation, which is generally considered less conservative than other horizontal preparation types such as a chamfer, or the vertical preparation like the knife‐edge design (Borelli et al. 2013; Florescu et al. 2026), despite being an effective design for all‐ceramic crowns (Shillingburg et al. 2012). The present findings are consistent with those of a previous study comparing knife‐edge and horizontal preparations (Monaco et al. 2019). However, the type of vertical preparation studied in that study was a knife‐edge preparation, which has a finish line, whereas the BOPT preparation studied in the current study has neither a finish line nor a margin. Furthermore, these results align with the findings of Real‐Voltas et al. (2025), which revealed that BOPT is more conservative in incisors and canines than both horizontal chamfer preparation and modified BOPT, the latter of which extends apically to the bone level (Real‐Voltas et al. 2025). However, the previous study revealed that premolars showed no difference among the three preparations, whereas a significant difference in tooth structure removal was observed between BOPT and shoulder preparation among all tooth types in the current study. Although the present findings are consistent with a recent systematic review which concluded that BOPT is more conservative than other subgingival preparations (Florescu et al. 2026), they contrast with the review's findings, which demonstrated that supragingival and juxta gingival chamfer preparations are more conservative. This variation could be attributed to differences between studies regarding the type of horizontal preparation; while the previous systematic review evaluated a chamfer preparation placed above the gingival margin for partial and full‐coverage restorations, the current study assessed a 1 mm rounded‐shoulder preparation located 0.5 mm coronal to the CEJ for full‐coverage crowns.

The findings showed that preparation for bilayered zirconia crowns resulted in 1.36 times more tooth structure removal compared with preparation for monolithic zirconia crowns. This may be attributed to the need for sufficient reduction to provide the required space for both the zirconia core and the veneering ceramic, whereas monolithic zirconia crowns achieve adequate strength with substantially less reduction (Mohaghegh et al. 2020). These results are consistent with those of Schwindling et al. (2019), who reported that monolithic zirconia crowns required the least amount of tooth structure removal in the central incisor group (42% by weight), compared with bilayered zirconia crowns and buccal veneered zirconia crowns (57% and 50%, respectively) (Schwindling et al. 2019). The results also align with the findings of another study that concluded that monolithic crowns, zirconia and lithium disilicate, were more conservative than metal‐ceramic crowns (Monaco et al. 2019). However, the present results do not correspond with those of Sadid‐Zadeh et al. (2018), who found that the amount of tooth structure removal for monolithic zirconia crowns was similar to that required for metal and lithium disilicate crowns (Sadid‐Zadeh et al. 2018). This discrepancy may be related to the fact that all three crown types evaluated in that study were monolithic, each fabricated from a single material. Additionally, their finding that monolithic lithium disilicate crowns required a similar structure removal to metal‐ceramic crowns contrasts with the current findings, which could be due to the different crown materials used in that earlier study (Sadid‐Zadeh et al. 2018).

When tooth type was taken into account, premolars showed the highest percentages of tooth structure removal by weight with no significant difference from central incisors. Conversely, molars and canines exhibited lower percentages, and the difference between them was also nonsignificant. However, the remaining pairwise comparisons demonstrated significant differences. These findings may be explained by variations in coronal dimensions and morphological characteristics among different tooth types. The crowns of both the central incisor and the canine exhibit a triangular shape; however, the canine is wider and thicker buccopalatally compared to the central incisor. Additionally, the narrow mesiodistal cervical width of the central incisor relative to its mesiodistal incisal width may increase its susceptibility to tooth structure loss during preparation compared to the canine and molar (Real‐Voltas et al. 2025). In contrast, the maxillary first molar is the largest tooth in the maxillary dental arch (Rikab 2011), which might provide greater resistance to extensive tooth structure removal due to preparation. However, the crown of the maxillary premolar has an oval shape that tapers toward the palatal cusp with a narrow mesiodistal width. These anatomical characteristics may explain why premolars demonstrated a greater susceptibility to tooth structure removal during preparation compared to molars and canines. These findings differ from the findings of a previous study which revealed that premolars were associated with the least structure removal compared with incisors and canines when modified BOPT and chamfer finish line were prepared (Real‐Voltas et al. 2025). That study also stated that BOPT‐prepared premolars exhibited similar tooth structure removal to BOPT‐prepared incisors and canines. This discrepancy could be attributed to differences in the preparation parameters used in the previous study; for instance, the chamfer was located 0.5 mm subgingivally. Moreover, premolars received 1 mm of axial reduction compared to 2 mm of palatal axial reduction in anterior teeth. Nonetheless, reductions were uniform in anterior and posterior teeth in the current study, and the shoulder was located 0.5 mm coronal to the CEJ, which might have influenced the findings, as the location of preparation margins may have important implications for dental structure preservation (Florescu et al. 2026). This finding is consistent with earlier studies reporting that premolars showed the greatest loss of tooth structure during preparation, followed by incisors and molars (Ebrahimpour and Toronto 2017; Florescu et al. 2026; Al‐Fouzan 2016). It also aligns with the conclusions of Weber et al. (2022), who concluded that tooth type influences the percentage of tooth structure removal regardless of the assessment method or restoration type (Weber et al. 2022). Although an earlier study evaluated preparations of incisors and molars, it compared different restoration types within each tooth category and did not include direct comparisons between tooth types (Schwindling et al. 2019).

The results also showed that horizontal preparation for bilayered zirconia crowns was associated with the greatest tooth structure removal, whereas the BOPT for monolithic crowns was the most conservative among the groups studied. These findings support the outcomes of the individual comparisons conducted in this study. Furthermore, they are in line with the results of a previous study demonstrating that vertical preparation for monolithic zirconia crowns was more conservative than conventional horizontal preparation for metal‐ceramic crowns (Monaco et al. 2019). However, the vertical preparation investigated in the previous study was a knife‐edge preparation rather than BOPT. It is important to note, however, that the effect of BOPT on tooth structure removal combined with different ceramic crowns has not been previously evaluated. These findings suggest that BOPT combined with monolithic zirconia crowns may provide a potential clinical option when conserving pulpal vitality is essential. Yet, the limitations of this *in vitro* study must be carefully considered before applying these results to clinical practice.

On the other hand, the volumetric and gravimetric assessment methods demonstrated a strong correlation. The gravimetric method was associated with higher values than the volumetric method. However, the values fell within the range reported in the literature (Monaco et al. 2019). The findings also revealed that both measurement methods might be used interchangeably to evaluate structural removal. These results could be explained by the use of the same tooth for both measurements. Moreover, all teeth were made of the same material, and therefore sharing the same physical properties, such as density and mass. This result is in line with a previous study (Monaco et al. 2019), which showed that there was an agreement between weight and volume assessments with differences ranging from 1% to 3%. Nevertheless, another study concluded that the assessment method did affect the evaluation of structure removal. This could be due to the methods used in that study (Weber et al. 2022); it assessed the weight and volume of prepared teeth after sectioning the roots of those teeth at the CEJ level. Moreover, the scanned coronal portions of the prepared teeth were milled and weighed. The milling process might include errors that affect the measurement. Also, the section level was located near the finish line which might have influenced the results, whereas sectioning in the current study was performed on additional teeth and far from the finish line or finish area.

One limitation of this study is that all preparations and assessments were performed by a single investigator, although the preparations were verified by an external examiner. Nonetheless, the operator was an expert in fixed prosthodontics, and was calibrated prior to the study. Moreover, sectioning the radicular part of the teeth may have resulted in the loss of cutting debris, which would not have been accounted for in the weight measurement (Monaco et al. 2019). Nevertheless, the section procedure was implemented on additional teeth and the correlation between the two assessment methods was strong. Taking advantage of the uniform properties and limited variables among acrylic teeth, the findings might differ from findings under clinical conditions due to the differences between natural teeth and acrylic teeth in physical properties, bur behavior, operator tactile feedback, and cutting efficiency. Therefore, the results should be interpreted with caution when extrapolating them to clinical practice. Furthermore, BOPT is a comprehensive clinical concept that consists of rotary curettage and the establishment of a new emergence profile. However, these clinical aspects were not represented under the conditions of this *in vitro* study. In addition, it is important to note that the horizontal preparation type assessed in this study was a rounded shoulder, which might be less conservative than a chamfer preparation (Borelli et al. 2013). Therefore, the current findings should be limited to comparisons of the preparation design in BOPT with the horizontal shoulder preparation. It is recommended to conduct a future *in vitro* study on extracted natural teeth to assess the influence of the BOPT preparation design on tooth structure removal compared to other types of vertical and horizontal preparation. Moreover, clinical trials might be suggested to investigate the effect of BOPT on structure removal under clinical conditions.

## 5 Conclusions

Within the limitations of this *in vitro* study using acrylic teeth, the following conclusions may be drawn: Preparation type and crown type significantly influenced the amount of tooth structure removal. The BOPT appears to offer a more conservative approach than a 1‐mm rounded‐shoulder horizontal preparation, particularly when combined with monolithic zirconia crowns. Tooth type also influences the extent of tooth structure removal during full‐crown preparation.

## Author Contributions

**Abeer Aljareh** conceived the study and initiated the study design, carried out the *in vitro* work, performed the gravimetric and volumetric analysis, analyzed and interpreted the data and drafted the manuscript. **Nabil Alhouri** participated in the conceptualization of the study, validated the data and tooth preparation, supervised the work, and reviewed and critically revised the manuscript. **Mohanad Kadhim** validated the data, supervised the work, and reviewed the manuscript.

## Funding

The authors have nothing to report.

## Ethics Statement

This study was approved by Damascus University Ethics Committee under code “DN‐261124‐353” on 26 November 2024.

## Conflicts of Interest

The authors declare no conflicts of interest.

## Data Availability

Data are available from the corresponding author upon reasonable request.
